# PD-L1: expression regulation

**DOI:** 10.1097/BS9.0000000000000149

**Published:** 2023-01-13

**Authors:** Yu-Jie Zhou, Guoli Li, Jiyin Wang, Mengyuan Liu, Zihan Wang, Yu Song, Xulong Zhang, Xi Wang

**Affiliations:** a Department of Immunology, School of Basic Medical Sciences, Capital Medical University, Beijing, China; b National Center for Infectious Diseases, Beijing Ditan Hospital, Capital Medical University, Beijing, China; c Beijing Key Laboratory of Emerging Infectious Diseases, Beijing Ditan Hospital, Capital Medical University, Beijing, China; d Beijing Institute of Infectious Diseases, Beijing, China; e Department of Medical Laboratory, Beijing Tongren Hospital, Capital Medical University, Beijing, China

**Keywords:** Drug discovery, Epigenetics, Immune checkpoint blockage, Immunotherapy, PD-L1 expression

## Abstract

Programmed death-ligand 1 (PD-L1), expressed on the surface of tumor cells, can bind to programmed cell death-1 (PD-1) on T cells. The interaction of PD-1 and PD-L1 can inhibit T-cell responses by decreasing T-cell activity and accelerating their apoptosis. Various cancers express high levels of PD-L1 and exploit PD-L1/PD-1 signaling to evade T-cell immunity, and immunotherapies targeting the PD-1/PD-L1 axis have been shown to exert remarkable anti-tumor effects; however, not all tumor patients benefit from these therapies. Therefore, study of the mechanisms regulating PD-L1 expression are imperative. In this review, we explore regulation of PD-L1 expression in the contexts of gene transcription, signaling pathways, histone modification and remodeling, microRNAs, long noncoding RNAs, and post-translational modification. Current developments in studies of agents that block PD-L1 and correlations between immunotherapies targeting PD-1/PD-L1 and PD-L1 expression are also summarized. Our review will assist in understanding of PD-L1 expression regulation and discusses the implications of reported findings in cancer diagnosis and immunotherapy.

## 1. INTRODUCTION

Cancer is a leading cause of death, killing millions of people annually. Lung, colorectal, and liver cancers are the most common types of malignancy. Over the past decade, immunotherapy has become a primary cancer treatment, alongside surgery, radiotherapy, and chemotherapy. Immune checkpoint blockade methods targeting cytotoxic-T-lymphocyte-antigen-4 (CTLA-4) or programmed cell death-1 (PD-1)/programmed death-ligand 1 (PD-L1) are currently being pursued by oncologists, because of their ability to treat cancer by restoring T-cell cytotoxicity against tumor cells.

PD-L1, also known as B7-H1 or CD274, was first characterized by Dong et al^1^ in 1999 and negatively regulates cellular immune responses. PD-L1 is the third member of the B7 family and a type I transmembrane protein comprising 290 amino acids. In 2000, Tasuku Honjo^2^ confirmed that PD-L1 is a ligand of PD-1. PD-L1 is expressed on various cells, including dendritic cells, lymphocytes, and endothelial cells. Tumor cells and their niches in many types of cancer, including melanoma, lung carcinoma, breast cancer, bladder cancer, pancreatic cancer, and ovarian cancer, can also express PD-L1.^3^ The engagement of T-cell PD-1 by PD-L1 expressed on tumor cells can inhibit the T-cell activation, negatively regulating adaptive immune responses, ultimately leading to tumor evasion of immunosurveillance and poor patient prognosis.

**Figure 1.**
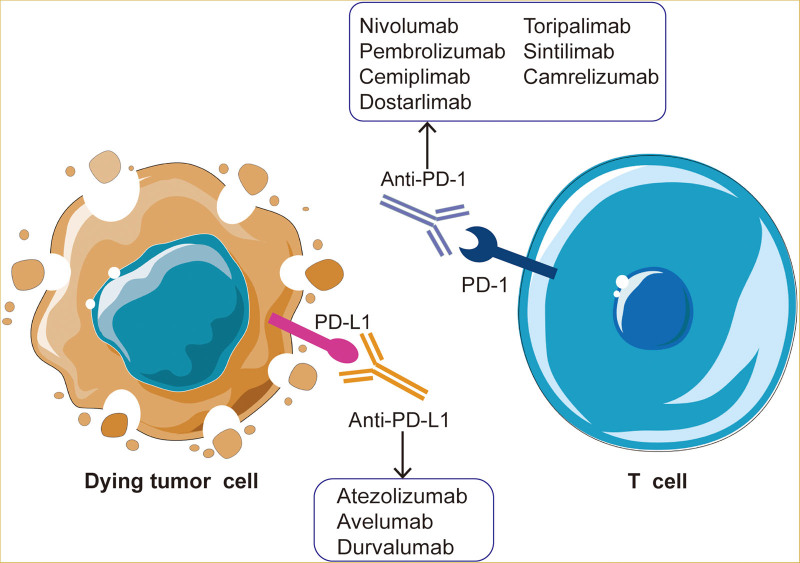
The enhancement effect of antibodies targeting PD-1 and PD-L1 on T cells killing tumor cells. PD-1 = programmed cell death-1, PD-L1 = programmed death-ligand 1.

Antibodies targeting PD-L1 can control immune escape and enhance adaptive immune responses, thereby killing tumor cells (**Fig. 1**). Some antibodies have been approved for clinical use by the U.S. Food and Drug Administration, including: atezolizumab (Tecentriq; Genentech, Inc.; DES MOINES, IA, USA) for triple negative breast cancer (TNBC), unresectable hepatocellular carcinoma (HCC), and extensive-stage small cell lung cancer; durvalumab (Imfinzi; AstraZeneca UK Limited, London, UK) for locally advanced or metastatic urothelial carcinoma; and avelumab (Bavencio; EMD Serono, Inc., Geneva, Switzerland) for advanced renal cell carcinoma.^4–8^ Currently, many antibodies against PD-L1 are under development or in clinical trials. Here, we review mechanisms of PD-L1 expression regulation and provide new ideas to inform development of immune checkpoint cancer therapy.

## 2. PD-L1 EXPRESSION IN DIFFERENT TUMOR STAGES

Various tumor cells can express PD-L1 to avoid killing by immune cells, and PD-L1 expression level can often determine the efficacy of immunotherapy against cancer. Whether PD-L1 expression varies significantly among different tumor stages and is important in tumor development remains unclear.

Nie et al^9^ found that PD-L1 expression level was correlated with cancer TNM (Tumor Node Metastasis) stage, but not with other clinical factors, including differentiation, cirrhosis, tumor size, or age, among others. Immunohistochemistry staining to determine PD-L1 expression level in lung adenocarcinoma was also correlated with TNM stage.^10^ Further, a meta-analysis by Xu et al^11^ showed that PD-L1 levels in tumor cells were correlated with cholangiocarcinoma TNM stage. Contrary to these reports, other research has found that PD-L1 expression level is not associated with tumor stage. Chen et al^12^ analyzed PD-L1 expression by immunohistochemistry in tumor and tumor adjacent-tissues from patients with gastric cancer, and found that PD-L1 was mainly expressed tumor cell membranes, but not in tissues adjacent to tumors. Furthermore, there was no correlation between PD-L1 and tumor stage. In non-*Schistosoma*-associated urinary bladder squamous cell carcinoma, PD-L1 expression was not related to T or N stages.^13^ Further, PD-L1 expression was an independent marker of clinical stage in nasopharyngeal carcinoma.^14^ In non-small cell lung cancer (NSCLC), PD-L1 levels were correlated with N, but not T or M stage.^15^ These data suggest that, in certain types of tumor, PD-L1 level may be related to tumor stage, and could be more significant only in certain stages. Whether PD-L1 expression in different tumor stages can be applied to the evaluation of immunotherapy warrants further study.

## 3. MONOCLONAL ANTIBODIES TARGETING PD-1/PD-L1 AND PD-L1 EXPRESSION

Whether tumors respond to therapeutic antibodies targeting the PD-1/PD-L1 axis depends on PD-L1 expression in tumor cells and their niches. In the research by Masuda et al^16^ into NSCLC with epidermal growth factor receptor (EGFR) mutation and high PD-L1 expression, PD-L1 expression level correlated with the efficacy of PD-1 inhibitors and could, therefore, be applied for assessment of PD-1 inhibitor efficacy. Bintrafusp alfa, a first-in-class bifunctional fusion protein that fuses the extracellular domain of transforming growth factor (TGF)-βRII receptor to human immunoglobulin G1 (IgG1) against PD-L1, can improve treatment efficacy in platinum-experienced patients with advanced NSCLC, particularly those with high PD-L1 expression on tumors.^17^ In a phase III clinical trial of atezolizumab, an anti-PD-L1 monoclonal antibody, on patients with nonsquamous or squamous metastatic NSCLC without prior chemotherapy, median survival of atezolizumab-experienced patients with tumors expressing wild-type EGFR, ALK (Anaplastic Lymphoma Kinase), and high levels of PD-L1 was 7.1 months longer than that of platinum-experienced patients.^18^ Further, PD-1/PD-L1 inhibitors are effective against pulmonary pleomorphic carcinoma, and longer progression-free survival (PFS) and overall survival (OS) are associated with higher patient PD-L1 expression.^19^ Hence, improved understanding of the mechanisms underlying PD-L1 expression regulation can inform drug selection for treatment of patients with cancer.

## 4. REGULATION OF PD-L1 AT THE GENETIC LEVEL

Oncogenesis is the result of a series of genomic changes,^20^ including gene rearrangements, gene loss, and specific copy number gains. Studies to date have revealed that genetic level aberrations can alter PD-L1 expression in cancer cells,^21^ thereby inducing their immune escape.

Genes encoding PD-L1 and PD-L2 map to chromosome 9p24.1, and are regulated by Janus kinase 2 (*JAK2*). Nodular sclerosing Hodgkin lymphoma is associated with an amplification of 9p24.1, which both directly promotes PD-L1 and PD-L2 expression and initiates transcription regulation of PD-1 ligands via JAK2.^22^ In a cohort of 125 cases of primary mediastinal large B-cell lymphomas, 20% of cases were identified with translocation and 29% with amplification of the PD-1 ligands locus at 9p24.1.^23^ Increasing evidence indicates that 9p24.1 amplifications are present in various tumors, including renal cell carcinomas with sarcomatoid transformation, TNBC, NSCLC, and gastric adenocarcinoma.^24–27^ Some types of classical Hodgkin lymphoma and primary central nervous system lymphoma respond well to nivolumab, because of copy number alterations at the PD-L1/PD-L2/JAK2 locus in tumors.^28,29^ Conversely, deletion of the *PD-L1* gene was detected in melanoma, lung adenocarcinoma, and lung squamous cell carcinoma,^30^ among other tumors, and could result in poor outcomes of treatment using PD-L1 inhibitors.

DNA double strand break (DSB) is a severe type of DNA damage, which can trigger DNA repair through phosphoinositol-3-kinase-related kinases, ataxia telangiectasia mutated, ataxia telangiectasia, and Rad3-related protein, and DNA-dependent protein kinase catalytic subunit.^31,32^ DSBs can be induced by ionizing radiation (IR),^33^ and PD-L1 is upregulated by IR. Furthermore, DNA damage activates the JAK1-signal transducer and activator of transcription 3 (STAT3) pathway, which promotes cancer cell survival and proliferation.^34^ Phosphorylation of STAT1/3 and Interferon Regulatory Factor 1 (IRF1) increases after DSB induction by IR, and loss of IRF1 significantly weakens PD-L1 upregulation following IR, suggesting that PD-L1 upregulation occurs via the STAT1/3-IRF1 pathway.^32^

Kataoka et al^35^ conducted a whole-genome sequencing study and demonstrated that disruption of the *PD-L1* 3′-untranslated region (UTR) promoted PD-L1 expression in various cancers. In addition, PD-L1 upregulation accompanied by 3′-UTR disruption depended on interferon gamma (IFN-γ) from T cells. The genomic variant, rs4143815, in the PD-L1 3′-UTR also significantly promotes PD-L1 expression in different cancers.^36,37^ By sequencing 276 gastrointestinal cancers, including colorectal, esophageal, HCC, gastric, and pancreatic cancers, Wang et al^36^ discovered that a G-C mutation in the PD-L1 3′-UTR could inhibit miR-570 binding and upregulate PD-L1 expression in gastric cancer. Additionally, the rs10815225 polymorphism in a binding site for the transcription factor, SP1, in the *PD-L1* promoter, is reported to upregulate *PD-L1* mRNA expression,^38^ indicating a different regulatory mechanism.

In conclusion, the studies cited above have shown that PD-L1 expression can be regulated at the genomic level. Further, the genomic alterations identified are potential biomarkers during patient diagnosis and prognosis, as well as in the clinical application of PD-1/PD-L1 checkpoint blockade.

## 5. SIGNALING PATHWAYS INVOLVED IN PD-L1 REGULATION

### 5.1. Inflammatory signaling pathways in PD-L1 regulation

Several inflammatory signaling pathways can regulate PD-L1 expression to maintain T lymphocyte immune tolerance via the PD-1/PD-L1 axis. Numerous inflammatory cytokines secreted from cells in the tumor microenvironment have vital roles in regulating PD-L1 expression.

#### 5.1.1. Type I and type II interferon

The type II interferon, IFN-γ, can be generated by activated T cells and NK (nature killer) cells, and is among the inflammatory cytokines most effective in promoting PD-L1 expression.^39^ The IFN-γ cell surface receptor comprises 2 subunits, IFNGR1 and IFNGR2, and IFN-γ binding couples the receptor with JAK1 and JAK2, which initiates phosphorylation of downstream STATs.^40,41^ IRF-1 is among the most important transcription factors in the IFN-γ signaling pathway, and activates PD-L1 by recognizing and binding to IRF-1 response elements (IRE1 and IRE2) in the *PD-L1* gene promoter in HCC cells.^42^

In addition to IFN-γ, the type I IFNs, IFN-α and IFN-β, can also regulate *PD-L1* gene transcription. The study by Bazhin et al^43^ confirmed that IFN-α regulates PD-L1 expression in dendritic cells via the STAT3 and p38 pathways. Moreover, IFN-β can mediate PD-L1 expression via JAKs- and IRF9-dependent or -independent pathways in mouse and human lung cancer cells,^44^ while both IFN-β and IFN-γ regulate PD-L2 expression in melanoma cells.^41^

### 5.2. Other inflammatory signaling

Both interleukin (IL)-17 and tumor necrosis factor α (TNF-α) can individually modulate *PD-L1* gene expression by activating AKT, nuclear factor-κB (NF-κB), and ERK (extracellular regulated protein kinases) 1/2 signaling in human colon cancer cells.^45^ Moreover, IL-17 promotes *PD-L1* expression in ovarian carcinoma.^46^ MAPK (mitogen-activated protein kinase) and JAK/STAT3 signaling inhibition suppresses PD-L1 expression simulated by IL-6 in thyroid cancer.^47^ Similarly, Zhang et al^48^ found that IL-6 could upregulate PD-L1 expression through JAK2/STAT1 and JAK2/STAT3/c-MYC signaling in monocytes and macrophages. An IL-20 antagonist can suppress PD-L1 expression in mouse pancreatic cancer, which could contribute to therapy for pancreatic ductal adenocarcinoma (PDAC) and cancer-associated cachexia.^49^ IL-27, which belongs to the IL-12 family, can active PD-L1 expression in human epithelial ovarian cancer cells by phosphorylating STAT1 and STAT3.^50^ PD-L1 expression can also be elevated in bladder cancer cells by chemokine (C-X-C motif) ligand (CXCL) 9 released from tumor-associated dendritic cells via CXCR3-related signaling.^51^ In addition, TNF-α can restore PD-L1 expression on lupus monocytes in vitro, while TGF-β has an opposite effect.^52^ Furthermore, David et al^53^ proved that TGF-β1 can initiate PD-L1 transcription via Smad (Mothers Against Decapentaplegic Homolog 2) phosphorylation in NSCLC.

### 5.3. Oncogenic signaling pathways

In addition to inflammatory signaling, multiple oncogenic pathways can also participate in PD-L1 expression regulation.

#### 5.3.1. PI3K/AKT signaling

Cell survival, proliferation, metabolism, and growth are dependent on the phosphatidylinositol-3-kinase (PI3K)/protein kinase B (PKB; also known as AKT) signaling. Dysregulation of the PI3K-AKT pathway increases the survival and proliferation of various cancer cells.^54^ PI3K activation converts phosphatidylinositol 4,5-bisphosphate (PI-4,5-P2) to phosphatidylinositol-3,4,5-trisphosphate (PI3,4,5-P3), and then recruits downstream AKT to modulate a series of cellular processes. mTORC1 can be activated by phosphorylated AKT and contribute to this process.^55^ AKT-mTOR (mammalian target of rapamycin) signaling pathway activation can upregulate PD-L1 expression in NSCLC.^56^ Moreover, PD-L1 expression can be increased via PI3K/AKT signaling, which is suppressed by the PI3K inhibitor, LY294002, in gastric cancer cell lines.^57^ As an important negative regulator of PI3K-AKT signaling, phosphatase and tensin homolog (PTEN) can promote the switch to PI-4,5-P2 from PI3,4,5-P3, while absence of PTEN can increase the PI3K-AKT activation in various tumors.^58^ Additionally, PTEN downregulation by specific siRNA in colorectal cancer increases PD-L1 levels.^59^

#### 5.3.2. MEK/ERK (MAPK) signaling

The Ras/Raf/MEK/ERK pathway is associated with cell survival, proliferation, and differentiation and is dysregulated in various tumor cells.^60^ MAPK plays a significant role in PD-L1 gene expression in lung adenocarcinoma. Suppression of the MAPK pathway using the MEK (MAPK ERK kinase) inhibitor, selumetinib, leads to reduced PD-L1 mRNA and protein induction by epidermal growth factor or IFN-γ.^61,62^ Activated MAPK signaling and increased PD-L1 expression, which can be blocked by MEK inhibitor, are often detected in BRAF (B-Raf proto-oncogene, serine/threonine kinase) inhibitor resistant melanoma cells.^63^ Similarly, in some pancreatic cancer cells, PD-L1 can be modulated by MAPK signaling, and this can be reversed by inhibiting MEK.^64^ Conversely, Loi et al^65^ studied breast cancer cell lines in vitro and in vivo and found that blocking MEK could positively regulate IFN-γ-mediated PD-L1 expression. Further, MAPK inhibitors (PD98059, UO126, and PD0325901) cannot prevent PD-L1 expression upregulation in the NKN7, KYSE30, or TE-1 cell lines.^66^

#### 5.3.3. MYC

MYC is a transcription factor with crucial roles in the cell, alongside MAPK and AKT, and MYC overexpression may contribute to tumorigenesis.^67–69^ MYC can directly bind to the *PD-L1* promotor, and MYC inactivation reduces PD-L1 expression.^70^ Analysis of a dataset from The Cancer Genome Atlas revealed a positive correlation between c-MYC and PD-L1 levels. Moreover, chromatin immunoprecipitation (ChIP) assays revealed that c-MYC the binding of to the *PD-L1* promoter resulted in elevated PD-L1 expression in esophageal squamous cell carcinoma (ESCC) cells.^71^ Atsaves et al^72^ found that PD-L1 was upregulated in response to MYC overexpression in anaplastic large-cell lymphoma; however, MYC deficiency may increase PD-L1 expression through IFN-γ-mediated STAT1 upregulation in HCC.^73^ MYC blockade using MYCi361 can enhance CD3^+^ T-cell infiltration and increase tumor growth, while elevating PD-L1 expression in the tumor microenvironment.^74^

#### 5.3.4. Signal transducer and activator of transcription 3

In response to type I IFNs (eg, IFN-γ and IL-6), STAT3 is phosphorylated, translocated, and can sequentially activate the transcription of various genes.^75–77^ In the absence of cytokine signaling, the STAT3 p.E616K mutant is phosphorylated and activated, and then binds to the *PD-L1* promoter to enhance PD-L1 expression in NK/T-cell lymphoma.^78^ In addition, ChIP assays demonstrated that STAT3 can bind directly to the *PD-L1* promoter in tolerogenic antigen-presenting cells.^79^ STAT3 activation increases PD-L1 expression and STAT3 inhibition by its suppressor results in PD-L1 downregulation in glioblastoma and castration-resistant prostate cancer.^80,81^

#### 5.3.5. Nuclear factor-kB

As a vital participant in cancer cell development, NF-κB can positively control PD-L1 expression in various cancers. PD-L1 expression can be upregulated through NF-κB activation in NSCLC with mutation in EGFR.^82,83^ Control of PD-L1 expression by NF-κB generally depends on inflammatory cytokines. Activation of NF-κB by IFN-γ or TNF-α can promote B7-H1 expression on myelodysplastic syndromes blasts.^84^ Additionally, activated NF-κB is involved in PD-L1 expression stimulated by lipopolysaccharide (LPS) in gastric cancer cells.^85^ There is conclusive evidence from several reports that binding of MYC and NF-κB p65 to the PD-L1 promoter enhanced PD-L1 expression via MUC1-C in TNBC, as well as by interferon-inducible 16 in cervical cancer cells and LPS-treated monocytes.^86–88^

#### 5.3.6. Hypoxia-inducible factor

Hypoxia is a crucial metabolic characteristic of tumors. Hypoxia-inducible factor-1 (HIF-1) is usually activated in tumor cells in the absence of oxygen and can regulate tumor progression and metastasis.^89^ Furthermore, under hypoxia, PD-L1 expression rises in a HIF-1α-dependent manner in human breast and prostate cancer cells, as well as in mouse melanoma and mammary carcinoma cells, which helps to increase their resistance to cytotoxic T lymphocytes.^90^ Data from chromatin immunoprecipitation and luciferase reporter assays show that HIF-1α binds directly to a hypoxia-response element in the *PD-L1* promoter, contributing to elevation of PD-L1 mRNA and protein levels in myeloid-derived suppressor cells.^91^ Further, PD-L1 expression decreased in response to HIF-2α silencing and reconstitution of pVHL in a study reported by Ruf et al^92^; hence, HIF-2α is involved in promotion of PD-L1 expression in clear cell renal cell carcinoma.

## 6. EPIGENETIC REGULATION OF PD-L1

### 6.1. Histone modifications

Covalent modifications of histones, such as acetylation, methylation, phosphorylation, and ubiquitination, play key roles in chromosome replication, gene transcription, and expression regulation, while alterations of histone modifications can often result in tumorigenesis.^93^

Histone acetylation characteristics have been widely studied in various cancer.^94^ Histone acetylation can be reversibly regulated by histone acetyltransferases (HATs) and histone deacetylases (HDACs).^95^ HDAC inhibitors can promote PD-L1 expression in anaplastic thyroid cancer and melanoma cells.^96^ Additionally, elevated PD-L1 was detected in the A549/CDDP, MCF7/ADR, and HepG2/ADR carcinoma cell lines, in which c-Jun was increased in response to reduced COP1 (E3 Ubiquitin Protein Ligase), whose accumulation can inhibit HDAC3, leading to a subsequent increase histone H3 acetylation around the *PD-L1* promoter, and promotion of PD-L1 expression.^97^ Another deacetylase, HDAC6, can also promote the PD-L1 expression by reducing histone acetylation close to *STAT3* in osteosarcoma cell lines and melanoma.^98,99^ Furthermore, HAT1 can positively regulate *PD-L1* transcription in PDAC.^100^

Histone methylation also influences PD-L1 expression regulation. In MCF-7 and BT-549 breast tumor spheres, the repressive histones, H3K9me3 and H3K27me3 bind weakly to the *PD-L1* promoter, while the positive regulatory histone, H3K4me3 binds strongly; and PD-L1 expression is mediated by histone methylation.^101^ Furthermore, PD-L1 expression can be initiated by the MLL1-H3K4me3 axis in pancreatic cancer^102^; H3K4me3 is enriched in the *PD-L1* proximal promoter in pancreatic cancer cells in vitro, while MLL1 is an H3K4 methylation-specific histone methyltransferase. ChIP analysis revealed that MLL1 could bind the *PD-L1* promoter and may directly stimulate *PD-L1* expression at the transcription level.^102^ Moreover, H3K4me3 levels flanking the *PD-L1* promoter region, as well as *PD-L1* expression, can be reduced by silencing MLL1 expression in pancreatic cancer cells.

### 6.2. Histone remodeling

In addition to histone modifications, folded chromatin structure can be altered by chromatin remodelers, which use energy from ATP (adenosine triphosphate) hydrolysis to slide, eject, or unwrap nucleosomes. Chromatin remodeling complexes comprise 4 families: switching defective/sucrose nonfermenting (SWI/SNF), imitation switch (ISWI), chromodomain, helicase, DNA binding (CHD), and inositol requiring 80 (INO80).^103^ SWI/SNF participates in nucleosome sliding and ejection, which fully exposes DNA, making it more accessible. AT-rich interaction domain 1A (ARID1A) is a prominent component of the SWI/SNF remodeling complex, and ARID1A deficiency results in elevated PD-L1 expression via PI3K/AKT signaling in HCC cells.^104^ Similarly, high PD-L1 levels were closely related to ARID1A deficiency in 273 patients with advanced gastric cancer. Flow cytometry, western blot, and qPCR (Quantitative Polymerase Chain Reaction) experiments demonstrated that membrane and total PD-L1 protein and *PD-L1* mRNA levels were upregulated by ARID1A blockade in vitro. Further analysis demonstrated that the PD-L1 expression was increased in response to ARID1A suppression through activating the AKT pathway.^57^ Fukumoto et al^105^ concluded that, by promoting binding of RNA polymerase II and H3K4me3 to the *PD-L1* promoter in ovarian cancer, ARID1A can suppress PD-L1 expression, both at physiological baseline and during inflammation with high IFN-γ.

## 7. MICRO RNA AND LONG NONCODING RNA REGULATION OF PD-L1

### 7.1. Modulation of PD-L1 expression by micro RNAs

Micro RNAs (MiRNAs) are endogenous noncoding RNAs comprising 19 to 25 bases that can downregulate target gene expression through pairing with the 3′-UTR of mRNA molecules. MicroRNAs play significant roles in various biological processes, and miRNA dysregulation contributes to diverse diseases, especially cancers. miRNAs have dual roles in various cancers, by inhibiting both onco-suppressive genes and oncogenes.^106^ PD-L1 expression is modulated directly and indirectly by miRNAs in various cancers. Dastmalchi et al^107^ demonstrated that miR-424-5p can regulate apoptosis and autophagy, as well as T-cell exhaustion, by increasing PD-L1 expression in breast cancer. The *PD-L1* mRNA 3′ UTR is directly bound by miR-513, thereby reducing IFN-γ-stimulated PD-L1 expression in cholangiocytes.^108^ Experiments using NSCLC cell lines and tissue samples from patients with NSCLC, and analysis of the miRNA target-predicting databases, demonstrated that *PD-L1* expression is directly suppressed by miR-34.^109^ Similarly, luciferase reporter assays have revealed that *PD-L1* is a target of miR-873 in breast cancer, miR-200c in TNBC, miRNA-148a-3p and miRNA-93-5p in colorectal cancer, and miR-138-5p in NSCLC.^110–114^

In addition to direct inhibition, there is extensive evidence that PD-L1 can be modulated indirectly by some miRNAs. Tang et al^115^ confirmed that miRNA-3127-5p could upregulate PD-L1 expression by stimulating its phosphorylation by STAT3, which promoted immune escape in NSCLC. In addition, miRNA-200a elevated PD-L1 expression in osteosarcoma cells through PTEN activity, which suppresses CD8^+^ T cell activation and induces tumor growth.^116^ Further, miR-375 negatively regulates PD-L1 via the JAK2/STAT3 axis in gastric cancer,^117^ and miR-27a-3p transported by exosome could remotely promote PD-L1 expression via MAGI2 (Membrane-associated guanylate kinase, WW and PDZ domain-containing protein 2)/PTEN signaling in breast cancer.^118^

### 7.2. Modulation of PD-L1 expression by long noncoding RNAs

Long noncoding RNAs (lncRNAs) can interfere with targeted miRNAs to regulate PD-L1 expression, and thereby affect the occurrence, development, and prognosis of tumors. Recent studies showed that PD-L1 expression is upregulated by lncRNA FGD5 antisense RNA 1 (*FGD5-AS1*) through suppression of miR-142-5P in ovarian cancer. Ultimately, *FGD5-AS1* accelerates tumor cell proliferation and metastasis,^119^ and enhances cisplatin resistance of lung adenocarcinoma, as well as facilitating lung adenocarcinoma progression via the miR-142-5P/PD-L1 axis.^120^ HIF-1α antisense RNA-2 (*HIF1A-AS2*)/miR-429^121^ and *MEG3*/miR-216a have become the focus of considerable researcher attention; both can positively regulate PD-L1 expression in aggressive endometrial cancer.^122^ LncRNA *EMX2OS* participated ovarian cancer formation and progression by mediating miR-654-3p/AKT3/PD-L1 signaling.^123^ Similarly, in vitro and in vivo experiments indicated that the lncRNA, *PSMB8-AS1*, directly targets miR-382-3p, which contributes to upregulation of PD-L1 expression in pancreatic cancer through enhancing STAT1 expression.^124^ Further, the lncRNA, *SNHG1*, is closely associated with PD-L1 induction in renal cell carcinoma via modulating the miR-129-3p/STAT3 axis, and could help tumor cells escape from immune responses.^125^ In conclusion, the essential roles of lncRNAs in PD-L1 expression modulation require corresponding miRNAs as mediators.

## 8. TRANSLATION MODIFICATION

Alternative splicing is a fundamental biological process in eukaryotes. After transcription, different exons of the initial RNA or RNA precursor are selectively reconnected through alternative splicing, to generate different splice isoforms. Thus, 1 gene can produce multiple different transcripts at different cell stages and in different tissues.^126^

There are multiple isoforms of the PD-L1 protein, all of which can play important roles in cancer progression and may be associated with responses to immunotherapy. Further, a lncRNA isoform of *PD-L1*, generated by alternative splicing, can promote lung adenocarcinoma progression, and the underlying mechanism involves enhancing c-MYC activity.^127^ Analysis of PD-L1 in peripheral blood mononuclear cells by He et al^128^ identified a novel PD-L1 isoform, with exon 2 spliced out. Unlike PD-L1, which is located on the surface of the cell membrane, this isoform is detected intracellularly because of the absence of the immunoglobulin variable domain-like domain. Among the 3 PD-L1 isoforms (isoform a, isoform b, and isoform c), isoform b inhibits T cells more effectively than isoforms a and c in colorectal cancer, suggesting that isoform b is a potential target for immunotherapy.^129^ These studies suggest that different PD-L1 isoforms are generated by alternative splicing and may play important roles in oncotherapy. The mechanisms regulating the different isoforms require further study, to identify more effective targets and biomarkers.

## 9. POST-TRANSLATIONAL MODIFICATION

After translation, newly-synthesized proteins are often inactive and require a series of post-translational modifications (PTMs) to become functional mature forms. PTMs refer to various chemical modifications of translated proteins, including: methylation, phosphorylation, glycosylation, acetylation, ubiquitination, deubiquitination, and palmitoylation. PTMs on specific amino acid residues can endow proteins with physical and chemical properties necessary for cell division, proteolysis, signal transduction, and protein-protein interactions. Many recent studies have shown that PD-L1 protein levels can be regulated by different PTMs during tumor growth and immunotherapy.^130^

### 9.1. Phosphorylation

Phosphorylation is among the best-studied PTMs and is involved in almost all cellular processes.^131^ Li et al^132^ reported that PD-L1 activity is regulated by phosphorylation, N-glycosylation, and ubiquitination. Glycogen synthase kinase 3β (GSK3β) phosphorylates the T180 and S184 residues of nonglycosylated PD-L1 and induces PD-L1 proteasome degradation through the β-TrCP (beta-transducin repeat-containing protein) pathway. EGFR activation can inhibit GSK3β, thereby maintaining PD-L1 stability.^133^ Another study proved that olaparib, a poly (ADP-ribose) polymerase inhibitor, could upregulate PD-L1 expression by suppressing GSK3β^134^; however, a recent study found that GSK3α, but not GSK3β, phosphorylates PD-L1 at Ser279/283, promoting its degradation. AMP-activated protein kinase, activated by metformin treatment for type 2 diabetes, could directly phosphorylate PD-L1 S195 and further glycosylate PD-L1,^135^ resulting in PD-L1 anergy. Meanwhile, abnormal PD-L1 accumulates in the endoplasmic reticulum (ER), which can lead to PD-L1 degradation through ER-associated degradation. Accordingly, phosphorylation at specific amino acids will cause PD-L1 degradation. Chan et al^136^ demonstrated that PD-L1 phosphorylated at residue Y112 through the IL-6/JAK1 pathway can recruit the N-glycosyltransferase, STT3A, to glycosylate PD-L1 and maintain its stability, which was inhibited by Y112F mutation. Hence, Y112-phosphorylation of PD-L1 represents a new PD-L1 phosphorylation modification that can promote PD-L1 stability.

### 9.2. Glycosylation

A glycosylated form of PD-L1 of approximately 45 kDa, with high activity, has been observed in cancer cells.^132^ Further, inhibitors of N-glycosylation, such as tunicamycin, swainsonine, castanospermine, and 1-deoxymannojirimycin, can reduce the affinity of PD-L1 binding to PD-1,^137^ indicating that N-glycosylation of PD-L1 is necessary for PD-L1 binding to PD-1. Meanwhile, inhibition of O-glycosylation mediated by B3GNT3 failed to disturb the PD-1/PD-L1 interaction, further supporting that N-glycosylation, rather than O-glycosylation, is the main glycosylation of PD-L1. During epithelial–mesenchymal transition, STT3 expression is enhanced, which promotes PD-L1 N-glycosylation.^138^ D’Arrigo et al^139^ reported that FKBP51s, a spliced isoform of FK506-binding protein of 51 kDa, is a co-chaperone of PD-L1 in the ER, and could upregulate PD-L1 expression by assisting the protein in folding into the specific structures necessary for N-glycosylation.

Together, the studies above confirm that N-glycosylation can stabilize PD-L1; therefore, glycosylated PD-L1 is a potential new target for drug design in immune checkpoint blockade therapy. Further, a recent study showed that the potential anti-cancer drug, Shikonin, could inhibit PD-L1 glycosylation via the NF-κB/STAT3 and NF-κB/CSN5 (COP9 signalosome subunit 5) pathways in pancreatic cancer cells.^140^ PD-L1 glycosylation may also impact its specific recognition by antibodies, thereby diminishing the effectiveness of monoclonal antibody drugs, and preventing diagnosis by disrupting antibody-dependent detection. Lee et al^141^ found that, when PD-L1 N-glycosylation was removed, the affinity of PD-L1 binding with its antibody was enhanced, resulting in improved pathological detection of PD-L1 and even benefiting anti-PD-1/PD-L1 therapy.

### 9.3. Ubiquitination

Ubiquitination, as another important post-translational modification of PD-L1, is necessary for protein degradation.^142^ CASP8 (Caspase8) promotes TNFAIP3 (TNF alpha induced protein 3) (A20) expression, leading to PD-L1 ubiquitination and degradation.^143^ In HCC cells, 2,5-dimethylcelecoxib promotes HBx (x protein of HBV)-induced PD-L1 ubiquitination via RBX1 (RING box protein-1), an E3 ubiquitin ligase.^144^ Besides, berberine (BBR) is a proven anti-inflammation drug that can enhance PD-L1 ubiquitination and promote its degradation by binding to CSN5 at Glu76 to inhibit its deubiquitination. Thus, some researchers in traditional medicine believe that BBR has potential as a treatment for NSCLC.^145^ Moreover, GATA binding protein 3 antisense RNA 1 (*GATA3-AS1*) promotes PD-L1 deubiquitination by miR-676-3p/COPS5 (CSN5) in human TNBC cell lines. Thus, *GATA3-AS1* can promote tumor progression by increasing PD-L1 stability.^146^ When GSK3α phosphorylates PD-L1, Ariadne-1 homolog (ARIH1), a member of the Ariadne family of E3 ubiquitin ligases, ubiquitinates PD-L1 via K48-linked ubiquitin chains, leading to PD-L1 degradation.^134^

### 9.4. Lipid modification

PD-L1 palmitoylation is a type of lipid modification, which can inhibit PD-L1 ubiquitination. Hence, PD-1 stability increases when a cysteine residue is bound covalently to palmitate by the zinc-finger DHHC-type-containing 3 (ZDHHC3) palmitoyl transferase, also referred to as DHHC9. When DHHC9 was competitively blocked, T cells regained their ability to kill breast tumor cells.^127,147^ Therefore, repressors of palmitoyl transferase have potential as adjuncts to immune checkpoint therapy.

## 10. CONCLUDING REMARKS

**Figure 2.**
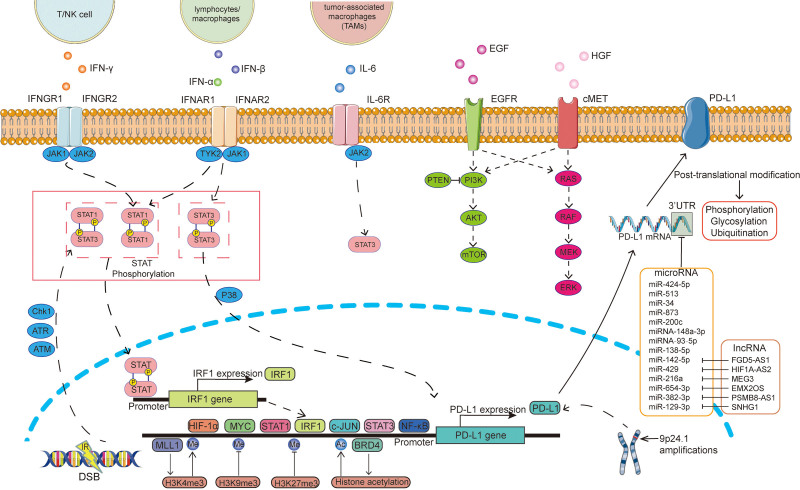
The regulatory mechanism of PD-L1 expression. PD-L1 = programmed death-ligand 1.

In this review, we focused on PD-L1 expression at different stages of cancer, as well as the relationship between PD-L1 expression and the therapeutic effects of PD-1/PD-L1 blockade in cancers. To this end, we explored the mechanisms regulating PD-L1 expression and summarized the current status of research into PD-L1 expression regulation at the genomic level, as part of signaling pathways, epigenetic regulation, miRNAs and lncRNAs, and post-translational modification (**Fig. 2**). The mechanisms regulating PD-L1 expression require further systematic study. The characteristics of PD-L1 in different tumor cells are highly heterogeneous. Thus, to develop personalized diagnosis and treatment procedures, it will be necessary to investigate PD-L1 expression in different types of cancer.

PD-L1 expression can also change after chemotherapy. Lacour et al^148^ found that PD-L1 expression increased in 35% patients with recurrent NSCLC after platinum-dependent chemotherapy; however, compared with the adjuvant chemotherapy group, PD-L1 expression was upregulated in only 12.5% cases of NSCLC with recurrence in the group without adjuvant chemotherapy, suggesting that the chemotherapy may increase PD-L1 expression. In another study, PD-L1 expression in ESCC cell lines also increased in vivo after treatment with 5-FU plus cisplatin or carboplatin plus paclitaxel. PD-L1 expression was upregulated via the EGFR/ERK pathway,^149^ suggesting that chemotherapy and anti-PD-L1 immunotherapy can be combined to achieve improved efficacy. Clinical trials of PD-1/PD-L1 antibodies in recent years are summarized in Table 1. In some clinical cases, anti-PD-L1 immunotherapy has been applied in combination with other monoclonal antibody drugs or chemotherapy against cancer. Planchard et al^195^ showed that OS and PFS of patients with metastatic NSCLC could be improved by treatment with durvalumab plus tremelimumab compared with the standard of care. In a phase 3 trial of patients with early-stage TNBC, atezolizumab plus chemotherapy could significantly improve pathological complete response rates compared with placebo plus chemotherapy.^168^ Similarly, atezolizumab combined with carboplatin and nab-paclitaxel could significantly improve PFS in patients with first-line squamous NSCLC. We believe that combination of monoclonal antibodies against the PD-1/PD-L1 axis with other drugs is promising as a treatment for tumors.

**Table 1:** Clinical trials related to PD-1 or PD-L1 in recent years.

Cancer type	Trial	Drugs	Target	Phase	References
Non-small cell lung cancer	EMPOWER-Lung 1	Cemiplimab	Cemiplimab: PD-1	3	^150^
Non-small cell lung cancer	CheckMate 9LA	Nivolumab plus ipilimumab combined with 2 cycles of chemotherapy	Nivolumab: PD-1; ipilimumab: CTLA-4	3	^151^
Squamous cell carcinoma of the head and neck	JAVELIN Head and Neck 100	Avelumab plus standard-of-care chemoradiotherapy vs chemoradiotherapy alone	Avelumab: PD-L1	3	^152^
Triple negative breast cancer	KEYNOTE-119	Pembrolizumab vs investigator-choice chemotherapy	Pembrolizumab: PD-1	3	^153^
Muscle-invasive urothelial carcinoma	IMvigor010	Adjuvant atezolizumab vs observation	Atezolizumab: PD-L1	3	^154^
Melanoma	EORTC 1325-MG/KEYNOTE-054	Adjuvant pembrolizumab vs placebo	Pembrolizumab: PD-1	3	^155^
NSCLC	CheckMate 227 Part 1	Nivolumab plus ipilimumab vs chemotherapy	Nivolumab: PD-1; ipilimumab: CTLA-4		^156^
Biliary tract carcinomas	IMMUNOBIL PRODIGE 57	Triplet combination of durvalumab, tremelimumab, and paclitaxel	Durvalumab: PD-L1; tremelimumab: CTLA4	2	^157^
Nonsquamous non-small cell lung cancer	CameL	Camrelizumab plus carboplatin and pemetrexed vs chemotherapy	Camrelizumab: PD-1	3	^158^
Non-small cell lung cancer	PEMBRO-RT and MDACC	Pembrolizumab with or without radiotherapy	Pembrolizumab: PD-1	2, 1/2	^159^
On-small cell lung cancer	POPLAR and OAK	Atezolizumab vs docetaxel	Atezolizumab: PD-L1	2, 3	^160^
Breast cancer	SAFIR02-BREAST IMMUNO	Durvalumab compared with maintenance chemotherapy	Durvalumab: PD-L1	2	^7^
Extensive-stage small-cell lung cancer	PASSION	Camrelizumab plus apatinib	Camrelizumab: PD-1	2	^161^
Relapsed small-cell lung cancer	CheckMate 331	Nivolumab vs standard chemotherapy	Nivolumab: PD-1	3	^162^
Relapsed or refractory classical Hodgkin lymphoma	KEYNOTE-204	Pembrolizumab vs brentuximab vedotin	Pembrolizumab: PD-1	3	^163^
Stage III melanoma	OpACIN-neo and OpACIN	Ipilimumab plus nivolumab	Nivolumab: PD-1; ipilimumab: CTLA-4	1b, 2	^164^
Non-small cell lung cancer	IMpower110	Atezolizumab	Atezolizumab: PD-L1	3	^18^
Non-small cell lung cancer	KEYNOTE-189	Pembrolizumab plus pemetrexed-platinum vs placebo plus pemetrexed-platinum	Pembrolizumab: PD-1	3	^165^
Advanced or metastatic urothelial carcinoma	JAVELIN Bladder 100	Maintenance therapy with avelumab plus best supportive care vs best supportive care alone	Avelumab: PD-L1	3	^166^
Unresectable, locally advanced or metastatic triple-negative breast cancer	IMpassion130	Atezolizumab plus nab-paclitaxel vs placebo-controlled	Atezolizumab: PD-L1	3	^4^
Advanced renal cell carcinoma	JAVELIN Renal 101	Avelumab plus axitinib vs sunitinib	Avelumab: PD-L1	3	^167^
Advanced non-small cell lung cancer	Merck KGaA	Bintrafusp alfa	Bintrafusp alfa: PD-L1&TGF-β	1	^17^
Early-stage triple-negative breast cancer	IMpassion031	Atezolizumab in combination with sequential nab-paclitaxel and anthracycline-based chemotherapy vs placebo and chemotherapy	Atezolizumab: PD-L1	3	^168^
Metastatic non-small cell lung cancer	MYSTIC	Durvalumab with or without tremelimumab vs standard chemotherapy	Durvalumab: PD-L1; tremelimumab: CTLA4	3	^169^
Previously untreated locally recurrent inoperable or metastatic triple-negative breast cancer	KEYNOTE-355	Pembrolizumab plus chemotherapy vs placebo plus chemotherapy	Pembrolizumab: PD-1	3	^170^
Advanced gastric cancer	KEYNOTE-062	Pembrolizumab or pembrolizumab plus chemotherapy vs chemotherapy alone	Pembrolizumab: PD-1	3	^171^
Advanced esophageal cancer	KEYNOTE-181	Pembrolizumab vs chemotherapy	Pembrolizumab: PD-1	3	^172^
Non-small cell lung cancer	IMpower131	Atezolizumab + carboplatin + paclitaxel, atezolizumab + carboplatin + nab-paclitaxel, or carboplatin + nab-paclitaxel	Atezolizumab: PD-L1	3	^173^
PD-L1–expressing advanced non-small cell lung cancer	KEYNOTE-010	Pembrolizumab vs docetaxel	Pembrolizumab: PD-1	2/3	^174^
Advanced gastric or gastroesophageal junction cancer	ATTRACTION-2	Nivolumab or placebo	Nivolumab: PD-1	3	^175^
Advanced renal cell carcinoma	JAVELIN Renal 101	Avelumab plus axitinib vs sunitinib	Avelumab: PD-L1	3	^176^
Non-small cell lung cancer	KEYNOTE-407	Pembrolizumab plus carboplatin and paclitaxel/nab-paclitaxel (chemotherapy) vs placebo plus chemotherapy	Pembrolizumab: PD-1	3	^177^
Metastatic castration-resistant prostate cancer	CheckMate 650	Nivolumab plus ipilimumab	Nivolumab: PD-1; ipilimumab: CTLA-4	2	^178^
Recurrent or metastatic head and neck squamous cell carcinoma	EAGLE	Durvalumab with or without tremelimumab	Durvalumab: PD-L1; tremelimumab: CTLA4	3	^179^
ER-positive breast cancer		Vorinostat, tamoxifen, and pembrolizumab	Pembrolizumab: PD-1	2	^180^
Non-small cell lung cancer	PEMBREIZH	Pembrolizumab	Pembrolizumab: PD-1		^181^
Resected stage IIIB–C and stage IV melanoma	CheckMate 238	Nivolumab vs ipilimumab	Nivolumab: PD-1; ipilimumab: CTLA-4	3	^182^
Previously untreated patients with unresectable, locally advanced or metastatic urothelial carcinoma	DANUBE	Durvalumab alone and durvalumab plus tremelimumab vs chemotherapy	Durvalumab: PD-L1; tremelimumab: CTLA4	3	^183^
Metastatic non-small cell lung cancer	KEYNOTE-024	Pembrolizumab vs chemotherapy	Pembrolizumab: PD-1	3	^184^
Advanced renal cell carcinoma	JAVELIN Renal 101	Avelumab plus axitinib vs sunitinib	Avelumab: PD-L1	3	^185^
Hormone receptor–positive, ERBB2-negative metastatic breast cancer		Eribulin with or without pembrolizumab	Pembrolizumab: PD-1	2	^186^
Advanced colorectal cancer	The Canadian Cancer Trials Group CO.26 Study	Tremelimumab and durvalumab plus best supportive care or best supportive care alone	Durvalumab: PD-L1; tremelimumab: CTLA4	2	^187^
Metastatic urothelial cancer	Hoosier Cancer Research Network GU14-182	Pembrolizumab vs placebo	Pembrolizumab: PD-1	2	^188^
High-risk stage III melanoma	EORTC 1325-MG/KEYNOTE-054	Pembrolizumab vs placebo	Pembrolizumab: PD-1	3	^189^
Recurrent or persistent ovarian cancer	NRG GY003	Nivolumab vs nivolumab and ipilimumab	Nivolumab: PD-1; ipilimumab: CTLA-4	2	^190^
Muscle-invasive bladder cancer	ENERGIZE	GC alone/combined with either nivolumab and linrodostat placebo/nivolumab plus linrodostat followed by RC and postsurgery continuation of immunotherapy	Nivolumab: PD-1; linrodostat: IDO1	3	^191^
Extensive-stage small-cell lung cancer	IMpower133	Atezolizumab, carboplatin, and etoposide	Atezolizumab: PD-L1	1/3	^192^
Metastatic, nonsquamous non-small cell lung cancer	KEYNOTE-189	Pembrolizumab or placebo plus pemetrexed and platinum	Pembrolizumab: PD-1	3	^193^
Advanced BRAF-mutant melanoma	COMBI-i	Spartalizumab plus dabrafenib and trametinib	Spartalizumab: PD-1; dabrafenib: BRAF; trametinib: MEK	3	^194^
Metastatic non-small cell lung cancer	ARCTIC	Durvalumab with or without tremelimumab	Durvalumab: PD-L1; tremelimumab: CTLA4	3	^195^
HER2-positive advanced breast cancer	KATE2	Trastuzumab emtansine plus atezolizumab vs trastuzumab emtansine plus placebo	Trastuzumab emtansine: HER2; atezolizumab: PD-L1	2	^196^
Locally advanced head and neck squamous cell carcinoma	KEYNOTE-412	Pembrolizumab or placebo	Pembrolizumab: PD-1	3	^197^
Metastatic renal cell carcinoma	IMmotion150	Atezolizumab alone or with bevacizumab vs sunitinib	Atezolizumab: PD-L1; bevacizumab: VEGF	2	^198^
Metastatic triple-negative breast cancer	ALICE	Atezolizumab combined with immunogenic chemotherapy	Atezolizumab: PD-L1	2	^199^
Advanced colorectal cancer	KEYNOTE-177	Pembrolizumab vs chemotherapy	Pembrolizumab: PD-1	3	^200^
MMR-proficient and MMR-deficient early-stage colon cancers	NICHE	Ipilimumab plus nivolumab, with or without celecoxib (pMMR)	Nivolumab: PD-1; ipilimumab: CTLA-4	2	^201^
Locally advanced or metastatic nonsquamous NSCLC		Sintilimab plus pemetrexed and platinum	Sintilimab: PD-1	3	^202^
Recurrent glioblastoma	CheckMate 143	Nivolumab vs bevacizumab	Nivolumab: PD-1; bevacizumab: VEGF	3	^203^
Advanced hepatocellular carcinoma		Camrelizumab	Camrelizumab: PD-1	2	^204^
Advanced or metastatic esophageal squamous cell carcinoma	ESCORT	Camrelizumab vs investigator’s choice of chemotherapy	Camrelizumab: PD-1	3	^205^
Untreated oral cavity squamous cell carcinoma		Nivolumab or nivolumab plus ipilimumab	Nivolumab: PD-1; ipilimumab: CTLA-4	2	^206^
Advanced pancreatic cancer	KEYNOTE144	Bruton tyrosine kinase inhibitor acalabrutinib, alone or with pembrolizumab	Pembrolizumab: PD-1	2	^207^
Advanced non-small cell lung cancer	JCOG1701	Atezolizumab/nivolumab/pembrolizumab	Atezolizumab: PD-L1; nivolumab: PD-1; pembrolizumab: PD-1	3	^208^
Locally advanced or metastatic non-small cell lung cancer	KEYNOTE-042	Pembrolizumab vs chemotherapy	Pembrolizumab: PD-1	3	^209^
Advanced non-small cell lung cancer	CheckMate 227	Nivolumab plus ipilimumab	Nivolumab: PD-1; ipilimumab: CTLA-4	3	^210^
Recurrent or metastatic squamous cell carcinoma of the head and neck	KEYNOTE-048	Pembrolizumab alone or with chemotherapy vs cetuximab with chemotherapy	Pembrolizumab: PD-1; cetuximab: EGFR	3	^211^
Advanced non-small cell lung cancer	KEYNOTE-024	Pembrolizumab vs platinum	Pembrolizumab: PD-1	3	^212^
Advanced renal-cell carcinoma	JAVELIN Renal 101	Avelumab plus axitinib vs sunitinib	Avelumab: PD-L1; axitinib: VEGFR	3	
Metastatic nonsquamous non-small cell lung cancer	IMpower130	Atezolizumab in combination with carboplatin plus nab-paclitaxel chemotherapy	Atezolizumab: PD-L1	3	^213^
Metastatic renal cell carcinoma	IMmotion151	Atezolizumab plus bevacizumab vs sunitinib	Atezolizumab: PD-L1; bevacizumab: VEGF	3	^214^
Advanced esophageal cancer	KEYNOTE-590	Chemotherapy with or without pembrolizumab	Pembrolizumab: PD-1	3	^215^
PD-L1–Low/negative recurrent or metastatic HNSCC	CONDOR	durvalumab with or without tremelimumab	Durvalumab: PD-L1; tremelimumab: CTLA4	2	^216^
Advanced esophageal squamous cell carcinoma	ATTRACTION-3	Nivolumab vs chemotherapy	Nivolumab: PD-1	3	^217^
Metastatic triple-negative breast cancer	TONIC	Nivolumab, irradiation, cyclophosphamide, cisplatin, doxorubicin	Nivolumab: PD-1	2	^218^
Early triple-negative breast cancer	GeparNuevo	Durvalumab in addition to an anthracycline taxane	Durvalumab: PD-L1	2	^219^
PD-L1-positive advanced non-small cell lung cancer	KEYNOTE-010	Pembrolizumab vs docetaxel	Pembrolizumab: PD-1	2/3	^220^
Advanced urothelial cancer	KEYNOTE-045	Pembrolizumab vs paclitaxel, docetaxel, or vinflunine	Pembrolizumab: PD-1	3	^221^
Advanced triple-negative breast cancer	IMpassion130	Atezolizumab plus nab-paclitaxel vs placebo plus nab-paclitaxel	Atezolizumab: PD-L1	3	^222^
Advanced non-small cell lung cancer	KEYNOTE-001	Pembrolizumab	Pembrolizumab: PD-1	1	^223^
Advanced non-small cell lung cancer	OAK	Atezolizumab vs docetaxel	Atezolizumab: PD-L1	3	^224^
Unresectable or metastatic melanoma	ECHO-301/KEYNOTE-252	Epacadostat plus pembrolizumab vs placebo plus pembrolizumab	Pembrolizumab: PD-1	3	^225^
Locally advanced head and neck cancer	JAVELIN Head and Neck 100	Avelumab and chemoradiation	Avelumab: PD-L1	3	^226^
Recurrent or metastatic squamous cell carcinoma of the head and neck	CheckMate 141	Nivolumab and cetuximab	Nivolumab: PD-1; cetuximab: EGFR	3	^227^
Metastatic melanoma	PCD4989g	Atezolizumab	Atezolizumab: PD-L1	1	^228^
Small cell lung cancer	IFCT-1603	Atezolizumab or chemotherapy	Atezolizumab: PD-L1	2	^229^
Gastric cancer	JAVELIN Gastric 100	Avelumab vs chemotherapy	Avelumab: PD-L1	3	^230^
PD-L1–expressing NSCLC	KEYNOTE-010	pembrolizumab vs docetaxel	Pembrolizumab: PD-1	2/3	^231^
Platinum-resistant recurrent ovarian cancer	KGOG 3045	Olaparib, cediranib, durvalumab, tremelimumab, and chemotherapy	Olaparib: PARP1/2; cediranib: VEGFR; durvalumab: PD-L1; tremelimumab: CTLA4		^232^
Relapsed/refractory classical Hodgkin lymphoma	S2016-127-01	Camrelizumab or decitabine plus camrelizumab	Camrelizumab: PD-1	2	^233^
Advanced esophageal cancer	KEYNOTE-590	Chemotherapy with or without pembrolizumab	Pembrolizumab: PD-1	3	^215^
BRAF-mutant melanoma		Dabrafenib, trametinib and pembrolizumab or placebo	Dabrafenib: BRAF; trametinib: MEK; pembrolizumab: PD-1	2	^234^
Advanced melanoma	KEYNOTE-006	Pembrolizumab vs ipilimumab	Pembrolizumab: PD-1; ipilimumab: CTLA-4	3	^235^
Relapsed malignant pleural mesothelioma	IFCT-1501 MAPS2	Nivolumab or nivolumab plus ipilimumab	Nivolumab: PD-1; ipilimumab: CTLA-4	2	^236^
Recurrent SCLC	CheckMate 032	Nivolumab	Nivolumab: PD-1	1/2	^237^
Sepsis		Nivolumab	Nivolumab: PD-1	1b	^238^
High-risk renal cell carcinoma	PROSPER RCC	Nivolumab	Nivolumab: PD-1	3	^239^
Advanced or metastatic renal cell carcinoma	PRISM	Nivolumab with ipilimumab	Nivolumab: PD-1; ipilimumab: CTLA-4	2	^240^

CTLA-4 = cytotoxic-T-lymphocyte-antigen-4, EGFR = epidermal growth factor receptor, ER = estrogen receptor, GC = gemcitabine plus cisplatin, HNSCC = head and neck squamous cell carcinoma, NSCLC = non-small cell lung cancer, PD-1 = programmed cell death-1, PD-L1 = programmed death-ligand 1, pMMR = mismatch repair-proficient, RC = radical cystectomy, TGF-β = transforming growth factor β, VEGF = vascular endothelial growth factor, VEGFR = vascular endothelial growth factor receptor.

Immunotherapy targeting the PD-L1/PD-1 axis has shown unprecedented efficacy in treating cancers. Nevertheless, only a proportion of patients can benefit from the treatment, although some do achieve complete recovery. Therefore, studies on the mechanisms regulating PD-L1 expression can both help to develop new drugs targeting PD-L1, including chemical compounds that inhibit PD-L1 checkpoint expression, to compensate for the inability of monoclonal antibodies to recognize PD-L1 as a consequence of PD-L1 modification or variation. In addition, PD-L1 expression checkpoints can also be used as biomarkers for detection during immune treatment, as an aid to drug selection and resistance evaluation.

Based on research into its regulation, we may also be able to develop methods to modulate PD-L1 expression on demand, which could enhance the efficacy of monoclonal antibody drugs against immune checkpoints in cancer therapy.

## ACKNOWLEDGMENTS

This work was supported by grants from the Support Project of High-level Teachers in Beijing Municipal Universities in the Period of 13th Five–year Plan (IDHT20190510) and the National Natural Science Foundation of China (81972652).

## AUTHOR CONTRIBUTIONS

Y.-J.Z. and G.L. contributed equally to this work.

Y.-J.Z., G.L., X.Z., and X.W. designed the article. Y.-J.Z. and G.L. searched the related literature and drafted the article. J.W., M.L. and Z.W. did literature searching and drafted several sections. Y.-J.Z. and Y.S. drew the figures and the table. X.Z. and X.W. revised the article. All authors contributed to the review and approved the final version.
